# Fc-engineered antibody therapeutics with improved efficacy against COVID-19

**DOI:** 10.21203/rs.3.rs-555612/v1

**Published:** 2021-05-27

**Authors:** Rachel Yamin, Andrew T Jones, Hans-Heinrich Hoffmann, Kevin S Kao, Rebecca L Francis, Timothy P Sheahan, Ralph S Baric, Charles M Rice, Jeffrey V Ravetch, Stylianos Bournazos

**Affiliations:** 1 Laboratory of Molecular Genetics and Immunology, The Rockefeller University, New York, NY; 2 Laboratory of Virology and Infectious Disease, The Rockefeller University, New York, NY; 3 Department of Epidemiology, University of North Carolina at Chapel Hill, Chapel Hill, NC, USA; 4 Department of Microbiology and Immunology, University of North Carolina at Chapel Hill, Chapel Hill, NC, USA

**Keywords:** Monoclonal Antibodies, Activating FcγRs, Fc Effector Function, Weight Loss and Mortality

## Abstract

Monoclonal antibodies (mAbs) with neutralizing activity against SARS-CoV-2 have demonstrated clinical benefit in cases of mild to moderate SARS-CoV-2 infection, substantially reducing the risk for hospitalization and severe disease^1–4^. Treatment generally requires the administration of high doses of these mAbs with limited efficacy in preventing disease complications or mortality among hospitalized COVID-19 patients^5^. Here we report the development and evaluation of Fc-optimized anti-SARS-CoV-2 mAbs with superior potency to prevent or treat COVID-19 disease. In several animal models of COVID-19 disease^6,7^, we demonstrate that selective engagement of activating FcγRs results in improved efficacy in both preventing and treating disease-induced weight loss and mortality, significantly reducing the dose required to confer full protection upon SARS-CoV-2 challenge and treatment of pre-infected animals. Our results highlight the importance of FcγR pathways in driving antibody-mediated antiviral immunity, while excluding any pathogenic or disease-enhancing effects of FcγR engagement of anti-SARS-CoV-2 antibodies upon infection. These findings have important implications for the development of Fc-engineered mAbs with optimal Fc effector function and improved clinical efficacy against COVID-19 disease.

Since the beginning of the COVID-19 pandemic, intense research efforts have focused on the isolation and clinical development of monoclonal antibodies (mAbs) that target the Spike protein of SARS-CoV-2 and exhibit potent neutralizing activity^4^. An ever-growing number of neutralizing anti-SARS-CoV-2 mAbs have entered clinical testing over the past few months, yielding FDA approval of two mAb cocktails –casirivimab and imdevimab (Regeneron) and bamlanivimab and etesevimab (Lilly)– for the treatment of patients with mild to moderate COVID-19 disease^4^. Results from large phase II/III studies have demonstrated that these and other mAbs that are currently awaiting regulatory approval offer a clear therapeutic benefit in patients with mild to moderate SARS-CoV-2 infection, reducing the risk for hospitalization and progression to severe COVID-19 disease by over 80%^1–3^. These results are in stark contrast to the findings from several phase III trials (e.g. ACTIV-3 protocols, NCT04501978) that assessed the therapeutic activity of these mAbs in hospitalized COVID-19 patients. In all cases, such trials failed to reach the primary study endpoints and were terminated prematurely, as none of the neutralizing anti-SARS-CoV-2 mAbs tested offered any therapeutic benefit over standard-of-care, even when administered at exceedingly high doses or in combination with antivirals like remdesivir^5^.

For several acute and chronic viral infections, including influenza virus, RSV, HIV, and Ebola virus, the antiviral activity of IgG antibodies is the outcome of Fab-mediated virus neutralization coupled with the capacity of the Fc domain to mediate effector functions through interactions with Fcγ receptors (FcγRs) expressed on the surface of effector leukocytes^8^. Indeed, FcγR engagement mediates pleiotropic antiviral immune functions, including the clearance of viral particles^9,10^, the cytotoxic elimination of virus-infected cells^11^, as well as the induction of protective T cell responses that contribute to antiviral immunity^12–14^. In the context of SARS-CoV-2 infection, a growing body of experimental evidence from various animal disease models supports that Fc- FcγR interactions are essential for the *in vivo* antiviral activity of anti-SARS-CoV-2 mAbs, as loss of the capacity of the Fc domain of these mAbs to engage FcγRs is associated with reduced antiviral activity *in vivo*^15–18^.

Given the importance of Fc-FcγR interactions in the mAb-mediated protection, maximizing the capacity of clinical neutralizing anti-SARS-CoV-2 mAbs to engage and activate the appropriate FcγR pathways is expected to lower the mAb dose required for the treatment of mild to moderate SARS-CoV-2 infection, as well as improve their therapeutic activity in hospitalized patients with severe COVID-19 disease. Currently, most mAbs in clinical use or development are expressed as human IgG1, which despite its affinity for activating FcγRs also exhibits considerable binding to the inhibitory FcγRIIb, thereby limiting protective Fc effector activities^9^. Additionally, several clinical mAbs (etesevimab, AZD8895, and AZD1061) lack Fc effector function, as they are expressed as Fc domain variants with no FcγR binding activity. This was due to presumptive safety concerns over the capacity of antibodies to exacerbate disease through ADE (antibody-dependent enhancement) mechanisms^8^. However, numerous *in vivo* studies in animal disease models have failed to provide evidence for ADE^15–19^, and therapeutic administration of high doses of convalescent plasma or neutralizing anti-SARS-CoV-2 mAbs in COVID-19 patients has not been associated with worse disease outcomes^1–3,5,20^. Likewise, comparable safety profiles were evident in clinical trials of neutralizing mAbs with intact or diminished Fc effector function.

To assess the role of FcγRs in the mAb-mediated protection and develop mAbs with superior therapeutic potency against COVID-19 disease, we selected well-established small animal SARS-CoV-2 infection models that recapitulate the clinical and pathological features of human COVID-19 disease^6,7,21^. One of these models involves the use of Syrian golden hamsters (*Mesocricetus auratus*), a species that not only sustains productive virus replication with SARS-CoV-2 clinical isolates, but also exhibits evidence of severe disease upon challenge^6^. However, a major obstacle in the *in vivo* study of Fc effector activity of human IgG antibodies is the substantial interspecies differences in the affinity of human IgG antibodies for FcγRs expressed by rodent species, such as hamsters^22^. We therefore cloned and expressed the four classes of hamster FcγRs and characterized their binding affinity for human, hamster, and mouse IgG subclasses and Fc variants (Fig. 1a–b, Extended Data Fig. 1). Comparative analysis of hamster FcγRs revealed substantial sequence homology to mouse FcγRs, with three hamster FcγRs (FcγRI, FcγRIII, FcγRIV) corresponding to activating FcγRs, whereas FcγRIIb represents the sole inhibitory FcγR.

To assess the contribution of Fc-FcγR interactions to mAb-mediated protection, we selected neutralizing mAbs in clinical use or development, including casirivimab and imdevimab (REGN cocktail^23^) and S309/VIR-7831 (Vir^24^) and expressed them as human IgG1 or as Fc domain variants with defined affinity for hamster FcγRs. In agreement with recent reports^16^, we observed that when mAbs are administered prophylactically, Fc effector function has minimal contribution to the mAb antiviral activity in this model (Fig. 1c). By contrast, in the therapy setting (d+1 treatment), wild-type, but not FcR null (GRLR) variants are able to suppress lung viremia and prevent weight loss (Fig. 1d). Since previous studies in mouse models of influenza virus and HIV-1 infection support a key role for FcγRIV in mediating protection by antiviral mAbs^11,25,26^, we compared the *in vivo* therapeutic activity of two Fc domain variants –GAALIE and V11– that exhibit differential hamster FcγRIV binding activity, but comparable affinity for the other hamster FcγRs (Fig. 1b). Consistent with a protective role for FcγRIV, the FcγRIV-enhanced variant (GAALIE) demonstrates potent antiviral activity, whereas no therapeutic activity is evident for V11, which exhibits minimal affinity for hamster FcγRIV (Fig. 1e–g).

Although these findings support the importance of Fc-FcγR interactions in the mAb-mediated protection against SARS-CoV-2 infection, their translational relevance is rather limited, given the diversity of FcγR expression on immune cells, the structural complexity of the FcγR family and the divergence of these receptors between humans and other mammalian species^22^. To address this problem, we have previously developed a mouse strain in which only human FcγRs are expressed in a pattern that recapitulates as faithfully as possible the expression pattern seen in human tissues^27^. Human FcγR expression among the various effector leukocyte populations is stable throughout mouse life span and does not differ between young and old mice (Extended Data Fig. 2). Infection of old (>15 weeks old), but not young (7 weeks), FcγR humanized mice with the mouse adapted SARS-CoV-2 strain MA10 (characterized in ^7^) results in rapid and often lethal weight loss, which is dependent upon the virus challenge dose (Fig. 2a–b). Histological evaluation of lungs from FcγR humanized mice (>15 weeks old) infected with MA10 revealed multifocal areas of interstitial pneumonia, extensive inflammatory cell infiltration, as well as evidence for occasional necrotic and proteinaceous material and hyalinized membranes within affected alveoli (Extended Data Fig. 3). Such inflammatory changes and histopathological findings are consistent with those observed in well-established animal models of SARS-CoV-2 infection used extensively in previous studies and resemble the lung pathology of human COVID-19 disease^7,15,16,18^.

In a model of mAb-mediated therapy of SARS-CoV-2 infection, we observed that the REGN mAb cocktail (expressed as wild-type human IgG1) confers full protection of FcγR humanized mice when administered at 5 mg/kg one day after lethal challenge with SARS-CoV-2 (MA10, 10^4^ pfu i.n.) (Fig. 2c–f). By contrast, no therapeutic activity is evident in mice lacking FcγRs (FcγR_null_) or when mAbs are expressed as variants (GRLR) with minimal affinity for human FcγRs, highlighting the importance of Fc effector function in the therapeutic activity of neutralizing mAbs (Fig. 2c–f). To determine the mechanisms by which human FcγRs contribute to the mAb-mediated protection, REGN cocktail mAbs were expressed as human IgG1 Fc variants that have been characterized extensively in previous studies^12,28^ and exhibit differential affinities for the various classes of human FcγRs (Fig. 3a). Following the experimental strategy outlined in Figure 3b, we assessed the therapeutic activity of Fc variants of the REGN mAb cocktail at a dose (1 mg/kg) which wild-type human IgG1 confers minimal protection (Fig. 3c). Consistent with a protective role for activating FcγRs, Fc variants enhanced for either FcγRIIa (GA) or FcγRIII (ALIE) show a trend for improved therapeutic potency over wild-type IgG1, whereas maximal therapeutic activity was evident only for the GAALIE variant, which is enhanced for both FcγRIIa and FcγRIII and has reduced affinity for the inhibitory FcγRIIb (Fig. 3d–e). These findings suggest that synergy between the two activating FcγRs, FcγRIIa and FcγRIII likely accounts for the therapeutic activity of the GAALIE variant, which achieves the same degree of protection as wild-type IgG1, but at a 5-times lower dose. Importantly, treatment of SARS-CoV-2-infected mice with the GAALIE variant was not associated with enhanced disease not only when administered at a low dose (1 mg/kg), but also at a much higher dose (40 mg/kg) (Extended Data Fig. 4). At this dose, which is typically used in the clinical setting, FcR null variants (GRLR) also exhibit full protective activity comparable to the GAALIE variants, suggesting that Fc-independent protection could be achieved once neutralizing mAbs are administered at sufficiently high doses, as has been documented previously for other viral pathogens^25,26^. Additionally, the observed differences in the *in vivo* therapeutic activity among Fc variants could not be attributed to differences in Fab-mediated functions, as none of these Fc modifications impact the *in vitro* neutralization activity, antigen binding specificity, or *in vivo* half-life (Extended Data Fig. 5–6).

Similar results were obtained when we assessed the *in vivo* therapeutic activity of another mAb cocktail (C135+C144; BMS/RU^29,30^) that is currently in clinical development and targets the SARS-CoV-2 Spike and mediates potent neutralizing activity^29^. In *in vivo* titration experiments, we observed that therapeutic administration of the BMS/RU mAb cocktail expressed as wild-type human IgG1 protects FcγR humanized from lethal SARS-CoV-2 challenge in a dose-dependent manner (Fig. 3f). When BMS-RU mAbs are administered to mice at 1 mg/kg, only GAALIE variants, but not wild-type human IgG1 confer protective activity and prevent disease-induced weight loss, confirming our findings on the REGN mAb cocktail, which demonstrated improved therapeutic activity when engineered for selective engagement of activating FcγRs (Fig. 3g).

Our findings in hamsters suggest that when neutralizing mAbs are administered prophylactically, Fc-FcγR interactions are not critical for their antiviral activity (Fig. 1c). However, given the substantial interspecies differences in FcγR biology between hamsters and humans, we assessed the contribution of activating FcγR engagement to the mAb-mediated prophylaxis of SARS-CoV-2-challenged FcγR humanized mice (Fig. 4a). When administered at a dose where wild-type human IgG1 exhibits no protective activity (0.5 mg/kg) (Fig. 4b), GAALIE variants of the REGN mAb confer full protection against lethal SARS-CoV-2 challenge, suggesting that selective activating FcγR engagement could improve the efficacy of neutralizing mAbs both at the therapeutic as well as at the prophylactic setting (Fig. 4c).

By coupling potent Fab-mediated neutralizing activity with pleiotropic Fc effector functions, neutralizing anti-SARS-CoV-2 mAbs confer antiviral protection and represent a key therapeutic modality for the control of COVID-19 disease in humans. Indeed, several recent reports have independently demonstrated in multiple well-defined *in vivo* experimental models of SARS-CoV-2 infection that the antiviral activity of neutralizing antibodies targeting the Spike protein of SARS-CoV-2 depends on Fc-FcγR interactions^15–18^. Additionally, mechanistic studies have determined that these protective effects are primarily mediated by FcγR-expressing, CCR2+ monocytes that infiltrate the lung and confer protective antiviral activities^16^. Despite these findings, no studies have explored whether optimization of neutralizing anti-SARS-CoV-2 mAbs for enhanced FcγR binding could improve their therapeutic activity, especially in the setting of severe COVID-19 disease.

To maximize the translational relevance of our findings, the present study focused exclusively on neutralizing anti-SARS-CoV-2 mAbs that are currently in clinical use or development and assessed their *in vivo* protective activity in mouse strains that recapitulate the unique complexity of human FcγRs^27^. Challenge of FcγR humanized mice (>15 weeks old) with mouse-adapted SARS-CoV-2 results in accelerated weight loss and significant mortality, resembling the clinical manifestations of severe COVID-19 disease that predominantly affects older human populations. In this clinically relevant experimental setting, we observed that in models of mAb-mediated prophylaxis or treatment, Fc engineering for selective binding to activating FcγRs results in approximately 5-fold reduction in the mAb dose required to achieve full therapeutic benefit; a finding consistent with our recent *in vivo* evaluation studies on Fc-optimized anti-influenza virus mAbs^12^.

Analysis of IgG Fc glycan characteristics in hospitalized COVID-19 patients has previously revealed an association of disease severity with elevated levels of afucosylated glycoforms^31,32^, which exhibit increased affinity for the activating FcγRIII^9^. Although these results suggest a potential pathogenic effect for FcγRIII pathways, severe COVID-19 patients are also characterized by delayed induction of neutralizing antibody responses and reduced serum neutralizing activity^33^, suggesting that any disease-enhancing effects of afucosylated antibodies might be related to their poor neutralizing activity. Indeed, studies in hamster and mouse models of SARS-CoV-2 infection using the non-neutralizing anti-SARS-CoV-2 mAb, CR3022 demonstrated that enhanced FcγR engagement is associated with increased pathology^34^. By contrast, in our study, we failed to observe any pathogenic or disease-enhancing effects upon administration of neutralizing anti-SARS-CoV-2 mAbs engineered for enhanced binding to activating FcγRs, highlighting the critical importance of Fab-mediated neutralization and Fc effector function during mAb-mediated protection.

Despite the successful deployment of highly effective vaccines, mAbs with potent neutralizing activity against SARS-CoV-2 are expected to continue to play an important role in virus containment efforts, as well as in the clinical management of COVID-19 disease, especially in high-risk populations and immunocompromised individuals. Although regulatory approval of anti-SARS-CoV-2 mAb cocktails has been based on their remarkable clinical efficacy in mild to moderate COVID-19 patients^1–3^, none of the currently approved mAbs take full advantage of the potential of an IgG molecule to mediate protective antiviral Fc effector functions. Our findings provide a paradigm for the development of mAb-based therapeutics with improved potency and superior therapeutic efficacy against COVID-19 through selective engagement of specific FcγR pathways.

## Methods

### Viruses, Cell Lines, and Animals

A P1 stock of the SARS-CoV-2 MA10 strain^7^ was amplified in VeroE6 cells obtained from the ATCC that were engineered to stably express TMPRSS2 (VeroE6_TMPRSS2_). To generate a P2 working stock, VeroE6_TMPRSS2_ cells were infected at a multiplicity of infection (MOI) of 0.1 plaque forming units (pfu)/cell and incubated at 37°C for 4 days. The virus-containing supernatant was subsequently harvested, clarified by centrifugation (3,000*g*; 10 min), and filtered using a disposable vacuum filter system with a 0.22 μm membrane. Virus stock titers were measured by standard plaque assay on Huh-7.5 cells that stably express ACE2 and TMPRSS2 (Huh-7.5_ACE2/TMPRSS2_). Briefly, 500 μl of serial 10-fold virus dilutions in Opti-MEM were used to infect 4×10^5^ cells seeded the day prior into wells of a 6-well plate. After 90 min adsorption, the virus inoculum was removed, and cells were overlayed with DMEM containing 10% FBS with 1.2% microcrystalline cellulose (Avicel). Cells were incubated for four days at 33°C, followed by fixation with 7% formaldehyde and crystal violet staining for plaque enumeration. To confirm virus identity and evaluate for unwanted mutations that were acquired during the amplification process, RNA from virus stocks was purified using TRIzol Reagent (ThermoFisher). Briefly, 200 μl of each virus stock was added to 800 μl TRIzol Reagent, followed by 200 μl chloroform, which was then centrifuged at 12,000*g* for 5 min. The upper aqueous phase was moved to a new tube, mixed with an equal volume of isopropanol, and then added to an RNeasy Mini Kit column (Qiagen) to be further purified following the manufacturer’s instructions. Viral stocks were subsequently confirmed via next generation sequencing using libraries for Illumina MiSeq.

The SARS-CoV-2 NYC isolate was obtained from a patient’s saliva generously provided by Agnès Viale (Memorial Sloan Kettering Cancer Center) and amplified in Caco-2 cells. This P1 virus was used to generate a P2 working stock by infecting Caco-2 cells at a MOI of 0.05 pfu/cell. Cells were incubated at 37°C for 6 days before harvesting virus-containing supernatant as described above. Similarly, virus stock titers were determined by plaque assay performed on Huh-7.5_ACE2/TMPRSS2_ cells. All SARS-CoV-2 experiments were carried out in biosafety level 3 (BSL-3) containment in compliance with institutional and federal guidelines.

VeroE6 cells (ATCC, CRL-1586), Caco-2 cells (ATCC, HTB-37), and Huh-7.5 hepatoma cells (described in ^35^) were cultured in Dulbecco’s Modified Eagle Medium (DMEM) supplemented with 1% nonessential amino acids (NEAA) and 10% fetal bovine serum (FBS). 293T cells (ATCC, CRL-3216) and HT1080_ACE2_ (kindly provided by Dr. Paul Bieniasz, Rockefeller University) were maintained in DMEM supplemented with 10% FBS, 50 U/ml penicillin and 50 μg/ml streptomycin (ThermoFisher). All cell lines were maintained at 37°C at 5% CO_2_. Expi293F cells (ThermoFisher) were maintained at 37°C, 8% CO_2_ in Expi293 expression medium (ThermoFisher) supplemented with 10 U/ml penicillin and 10 μg/ml streptomycin. All cell lines have been tested negative for mycoplasma contamination.

*In vivo* experiments were approved by the Rockefeller University Institutional Animal Care and Use Committee in compliance with federal laws and institutional guidelines. Hamsters and mice were maintained at the Comparative Bioscience Center at the Rockefeller University at a controlled ambient temperature environment with 12-h dark/light cycle. Golden Syrian hamsters were purchased from Charles River laboratories (strain code 049) and maintained in compliance with USDA regulations. FcγR knockout (mFcγRα^−/−^; *Fcgr1*^−/−^) and FcγR humanized mice (mFcγRα^−/−^, *Fcgr1*^−/−^, hFcγRI^+^, hFcγRIIa_R131_^+^, hFcγRIIb^+^, hFcγRIIIa_F158_^+^, and hFcγRIIIb^+^) were generated in the C57BL/6 background and characterized in previous studies^12,27^.

### Cloning, Expression, and Purification of Recombinant Proteins

Human IgG1 Fc domain variants were generated by site-directed mutagenesis using specific primers as previously described^10^ and recombinant IgG antibodies were expressed and purified using previously described protocols^12^. Purity was assessed by SDS–PAGE followed by SafeStain blue staining (ThermoFisher). All antibody preparations were more than 90% pure and endotoxin levels were less than 0.05 EU/mg, as measured by the limulus amebocyte lysate assay.

The two plasmid-based HIV/NanoLuc-SARS-CoV-2 pseudovirus system was kindly provided by Dr. Paul Bieniasz (described in ^36^). The S gene was modified by side-directed mutagenesis to introduce the amino acid changes present in the MA10 strain^7^. SARS-CoV-2_MA10_ pseudovirus particles were generated by transfection of the two plasmid-based system to 293T cells using X-tremeGENE HP DNA transfection reagent (Sigma).

Golden Syrian hamster FcγR cDNA sequences were identified based on the current *Mesocricetus auratus* genome assembly (MesAur1.0) and recent transcriptomic data^37^. To validate sequences, hamster FcγR sequences were amplified and sequenced (Genewiz) from Syrian hamster spleen cDNA (Zyagen) using the following primers: hamster FcγRI (5’-GGC GGA CAA GTG GTA AAT GTC AC-3’, 5’-CGG ACA CAT CAT TGC TTC AGA CTT ACT AAG-3’), hamster FcγRII (5’-CTG CTG GGA CAC ATG ATC TCC-3’, 5’-TTA AAT GTG GTT CTG GTA ATC ATG CTC TG-3’), hamster FcγRIII (5’-GAG TCT GGA GAC ACA GAT GTT TCA G-3’, 5’-CGA CGT CAT TTG TCC CGA GG-3’), hamster FcγRIV (5’-AAT GGG TGA GGG TGC TTG AG-3’, 5’-GAG GGA ATG TTG GGG ACA GG-3’). To identify the ectodomain, transmembrane, and cytoplasmic domains, Syrian hamster FcγR protein sequences were then aligned against annotated mouse FcγR protein sequences (Uniprot). Soluble Syrian hamster FcγR ectodomains were generated by transient transfection as described above for mAbs. Syrian hamster FcγR expression plasmids were generated encoding a secretion signal peptide, the predicted Syrian hamster FcγR ectodomain, and a C-terminal Strep or His tag. Following transfection of Expi293 cells, recombinant FcγRs were purified from cell-free supernatants by affinity purification using Strep-Tactin Superflow Plus resin (Qiagen) or Ni-NTA Agarose (Qiagen), per manufacturer’s instructions. Purified proteins were dialyzed into PBS and assessed for purity by SDS-PAGE gel electrophoresis followed by SafeStain blue staining. Monomeric FcγR ectodomains were fractionated from aggregates by size exclusion chromatography on an Äkta Pure system using a Superdex 200 Increase 10/300 GL column (GE Healthcare).

### Surface Plasmon Resonance

All experiments were performed with a Biacore T200 SPR system (GE Healthcare) at 25 °C in HBS-EP^+^ buffer (10 mM HEPES, pH 7.4, 150 mM NaCl, 3.4 mM EDTA, 0.005% (v/v) surfactant P20). IgG antibodies were immobilized on Series S Protein G sensor chip (GE Healthcare) at a density of 2,000 response units (RU). Serial dilutions of recombinant soluble hamster FcγR ectodomains were injected to the flow cells at 20 μl/min, with the concentration ranging from 1000 to 15.625 nM (1:2 successive dilutions). Association time was 60 s followed by a 900-s dissociation step. At the end of each cycle, sensor surface was regenerated with a glycine HCl buffer (10 mM, pH 2.0; 50 μl/min, 30 s). Background binding to blank immobilized flow cells was subtracted, and affinity constants were calculated using BIAcore T200 evaluation software v.2.0 (GE Healthcare) using the 1:1 Langmuir binding model.

### Neutralization Assay

Neutralization activity of IgG1 Fc domain variants was measured as previously described^36^. Briefly, HT1080_ACE2_ cells were seeded in 96 U-well plates 24 h prior to infection with SARS-CoV-2_MA10_ pseudoviruses. Pseudovirus particles were pre-incubated with mAbs (four-fold serially diluted starting at 10 μg/ml) for 1 h at 37°C and then added to a monolayer of HT1080_ACE2_ cells. Following a 48-h incubation at 37°C, cells were carefully washed with PBS and lysed with Luciferase Cell Culture Lysis reagent (Promega) for 15 min. Nano luciferase activity was detected by adding Nano-Glo Luciferase Assay System (Promega) and measured by SpectraMax Plus spectrophotometer (Molecular Devices), using 0.5 s integration time. Data were collected and analyzed using SoftMax Pro v.7.0.2 software (Molecular Devices). Relative luciferase units were normalized to those derived from cells infected with SARS-CoV-2_MA10_ pseudoviruses in the absence of mAbs.

### Anti-SARS-CoV-2 RBD ELISA

Recombinant SARS-CoV-2 RBD was immobilized (1 μg/ml) into high-binding 96-well microtitre plates (Nunc) and after overnight incubation at 4 °C, plates were blocked with PBS plus 2% (w/v) BSA for 2 h. After blocking, plates were incubated for 1 h with serially diluted IgG antibodies or serum samples (1:3 consecutive dilutions in PBS starting at 100 ng/ml), followed by HRP-conjugated goat anti-human IgG (1 h; 1:5,000; Jackson Immunoresearch). Plates were developed using the TMB two-component peroxidase substrate kit (KPL) and reactions were stopped with the addition of 1 M phosphoric acid. Absorbance at 450 nm was immediately recorded using a SpectraMax Plus spectrophotometer (Molecular Devices) and background absorbance from negative control samples was subtracted. Data were collected and analysed using SoftMax Pro v.7.0.2 software (Molecular Devices).

### Quantification of Serum IgG Levels

Blood was collected into microvette serum gel tubes (Sarstedt) and serum was fractionated by centrifugation (10,000*g*, 5 min). IgG levels were determined by ELISA following previously described protocols^12^.

### In vivo SARS-CoV-2 Infection Models

All animal infection experiments were performed at the Comparative Bioscience Center of the Rockefeller University in animal biosafety level 3 (ABSL-3) containment in compliance with institutional and federal guidelines. Hamsters (males; 6–8 weeks old) were anaesthetized with isoflurane (3%) in a VetFlo high-flow vaporizer followed by an intraperitoneal injection of a ketamine (150 mg/kg) and xylazine (10 mg/kg) mixture. Hamsters were challenged intranasally with 10^5^ pfu SARS-CoV-2 (NYC isolate, 100 μl viral inoculum). Mice (males or females; 6–7 or 16–22 weeks old) were anaesthetized with a ketamine (75 mg/kg) and xylazine (15 mg/kg) mixture (administered intraperitoneally) prior to challenge with SARS-CoV-2 (MA10 strain, 10^4^ pfu in 30 μl, intranasally).

After infection, animals were monitored daily and humanely euthanized by CO_2_ asphyxiation at endpoints authorized by the Rockefeller University Institutional Animal Care and Use Committee, including any of the following: marked lethargy or inactivity, severe respiratory distress or labored breathing, inability to ambulate, and weight loss of greater than 20% of baseline. Animals were randomized based on age, gender, and weight. Before treatment, we ensured that the mean weight, gender, and age were comparable among the various treatment groups. For antibody-mediated prophylaxis, antibodies were administered intravenously one day before virus challenge, whereas for antibody-mediated therapy, antibodies were administered one day after infection. Antibody dose was calculated as mg/kg.

### Histological Analysis

Lungs from euthanized mice were instilled with 10% neutral buffered formalin and fixed overnight by submersion in 10% formalin. Fixed tissues were embedded in paraffin, sectioned at 4 μm thickness, and stained with hematoxylin and eosin. Sections of lung were microscopically evaluated by a board-certified veterinary anatomic pathologist and representative images were captured with an Olympus BX45 light microscope using an SC30 camera with the cellSens Dimension software.

### Determination of Lung Viral Titers

Hamsters were euthanized at the indicated timepoints following infection and lung weights were recorded. Lungs were lysed in Trizol (ThermoFisher) and dissociated in gentle MACS M tubes using the gentleMACS Octo Dissociator (Miltenyi Biotec). Samples were transferred into Phasemaker tubes (ThermoFisher) and chloroform was added (200 μl chloroform/1 ml TRIzol). After vigorous shake, tubes were rested for 5 min and then centrifuged for 15 min at 12,000*g* at 4°C. The aqueous phase containing the RNA was transferred into a new tube and RNA extraction was performed by using RNeasy mini kit (Qiagen). SARS-CoV-2 lung titers were determined by qRT-PCR assay using TaqMan Fast Virus 1-Step Master Mix and specifically designed primers and a TaqMan probe that bind a conserved region in the nucleocapsid gene of SARS-CoV-2 (2019-nCoV_N1-F :5’- GAC CCC AAA ATC AGC GAA AT-3’. 2019-nCoV_N1-R: 5’- TCT GGT TAC TGC CAG TTG AAT CTG-3’. 2019-nCoV_N1-P: 5’- FAM-ACC CCG CAT TAC GTT TGG TGG ACC-BHQ1-BHQ1–3’). qPCR was performed using an Applied Biosystems QuantStudio 6 Flex cycler using the following parameters: 50°C for 5 min, 95°C for 20 s followed by 40 cycles of 95°C for 3 s, and 30 s at 60°C. Signal from unknown samples was compared to a known standard curve (obtained through BEI Resources, NIAID, NR-52358) and viral titers were expressed as RNA copies/mg tissue.

### Flow Cytometry

After lysis of red blood cells (RBC lysis buffer; Biolegend), cells were resuspended in PBS containing 0.5% (w/v) BSA and 5 mM EDTA and labelled with the following fluorescently labelled antibodies (all used at 1:200 dilution unless otherwise stated): anti-human FcγRIIa (clone IV.3)-FITC, anti-human FcγRIIb (clone 2B6)-Dylight650 (used at 5 μg/ml), anti-B220-AlexaFluor700, anti-Gr1-BrilliantViolet421, anti-CD8β-BrilliantViolet510, anti-human FcγRI (clone 10.1)-BrilliantViolet605, anti-CD3-BrilliantViolet650 (used at 1:100 dilution), anti-CD11b-BrilliantViolet711, anti-CD4-BrilliantViolet785, anti-human FcγRIIIa/b (clone 3G8)-PE, and anti-NK1.1-PE/Cy7. Relevant isotype control antibodies were used and included: mouse IgG1 isotype control-Dylight650 (used at 5 μg/ml), mouse IgG2b kappa isotype control-FITC, mouse IgG1 kappa isotype control-PE, mouse IgG1 kappa isotype control-BrilliantViolet605. Samples that were stained with isotype control antibodies were also blocked with unlabeled anti-FcγR antibodies as follow: anti-human FcγRI (clone 10.1), anti-human FcγRIIa (clone IV.3), anti-human FcγRIIb (clone 2B6), and anti-human FcγRIIIa/b (clone 3G8) (used at 10 μg/ml and incubated for 5 min prior to staining with fluorescently labelled antibodies). Samples were analyzed on an Attune NxT flow cytometer (ThermoFisher) using Attune NxT software v3.1.2 and data were analyzed using FlowJo (v10.7) software.

### Statistical Analysis

Results from multiple experiments are presented as mean ± s.e.m. One- or two-way ANOVA was used to test for differences in the mean values of quantitative variables, and where statistically significant effects were found, post hoc analysis using Bonferroni (adjusted for multiple comparisons) test was performed. Statistical differences between survival rates were analyzed by comparing Kaplan–Meier curves using the log-rank (Mantel–Cox) test. Data were analyzed with GraphPad Prism v.9.1 software (GraphPad) and p < 0.05 were considered to be statistically significant.

## Extended Data

**Extended Data Fig. 1:**
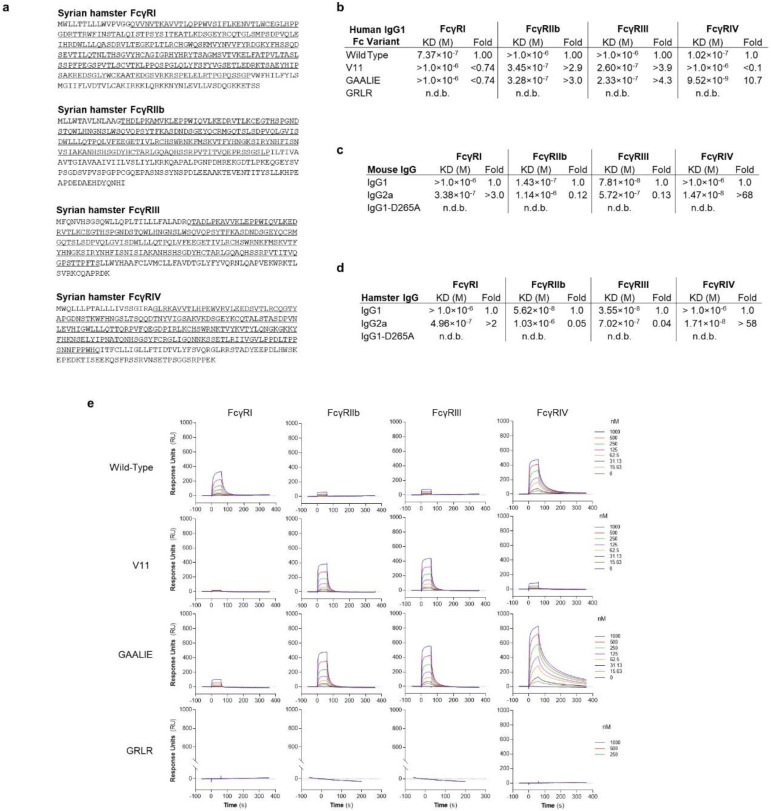
Cloning and characterization of the IgG binding activity of hamster FcγRs. **a**, Syrian hamster FcγRs were cloned, and their sequences were determined. The FcγR ectodomains are underlined. **b-e**, The affinity of human IgG1 and Fc variants (**b**, **e**, SPR sensorgrams), as well as of mouse (**c**) and hamster (**d**) IgG subclass variants for the various classes of hamster FcγRs was determined by surface plasmon resonance (SPR), using soluble hamster FcγR ectodomains. n.d.b., no detectable binding.

**Extended Data Fig. 2:**
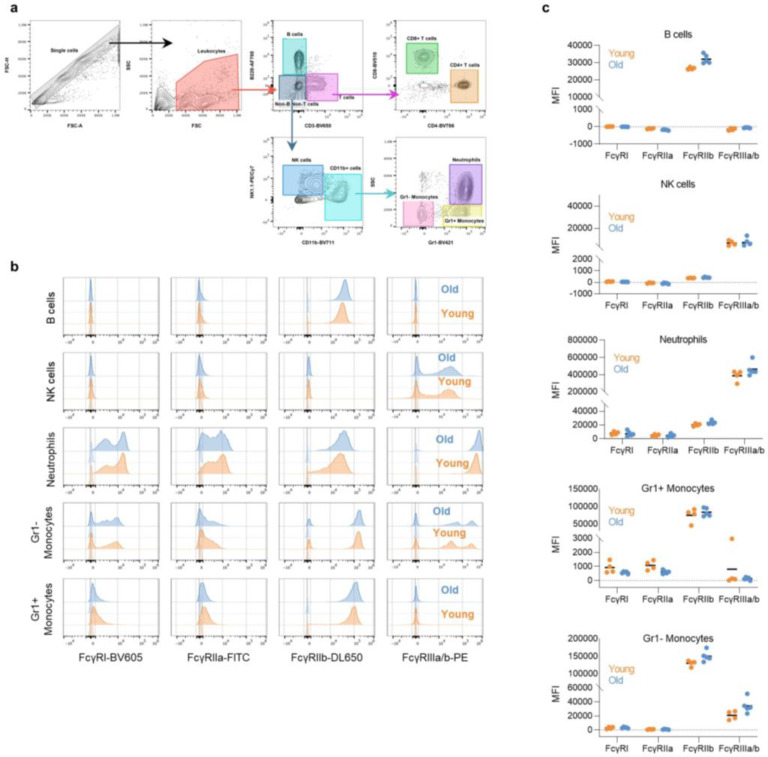
Comparison of the FcγR expression levels in the various effector leukocyte populations between young and older FcγR humanized mice. FcγR expression was assessed by flow cytometry in peripheral blood leukocyte populations from young (6–7 weeks old; orange) and older (17 weeks old; blue) FcγR humanized mice. **a**, Gating strategy to identify the various leukocyte populations, **b**, Representative histogram overlay plots of FcγR expression in young and older FcγR humanized mice. Corresponding isotype controls are indicated in lighter shade. **c**, Quantitation of FcγR expression (MFI, median fluorescence intensity subtracted from the respective isotype control) in various leukocyte populations. Results are from 4 or 5 mice per group for young and older mice, respectively.

**Extended Data Fig. 3:**
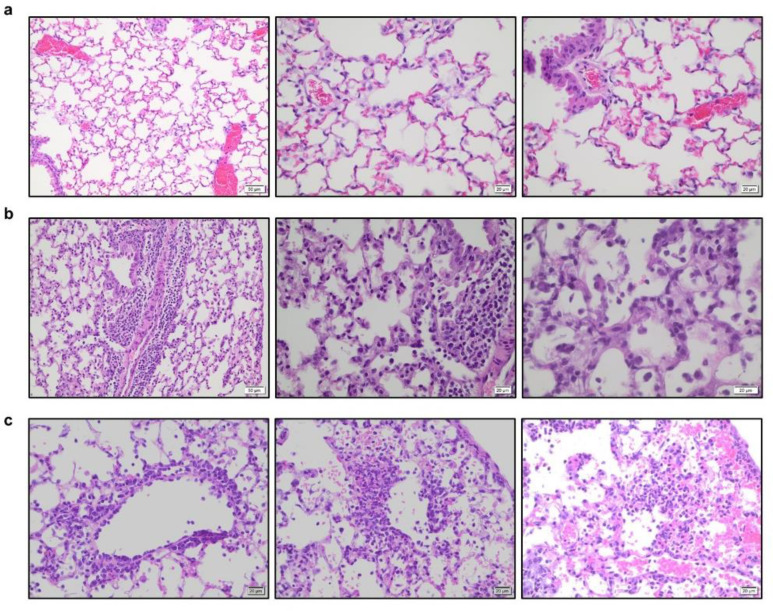
Histopathological analysis of lung tissue from SARS-CoV-2-infected FcγR humanized mice. Lungs from SARS-CoV-2-infected (MA10 strain, 10^4^ pfu, i.n.) FcγR humanized mice (16–22 weeks old) were harvested on day 4 post-infection and evaluated histologically to assess the pathological changes associated with SARS-CoV-2 infection. **a**, Uninfected mice were characterized by clear alveolar spaces and absence of inflammatory cell infiltrates (low magnification (left panel, 200x); high magnification (center and right panels, 400x). **b**, In contrast, SARS-CoV-2 infection was associated with perivascular and peribronchial mononuclear leukocyte infiltration (400x, left and center panels), as well as the presence of macrophages and neutrophils in alveoli and necrotic cellular debris in alveolar spaces (600x, right panel). **c**, In addition, SARS-CoV-2-infected mice exhibited perivenular mixed neutrophilic, histiocytic, and lymphocytic inflammation, reactive endothelium and extravasation of leukocytes (left panel, 400x), as well as foci of interstitial neutrophilic and macrophage inflammation with hemorrhage and single cell necrosis (center and right panels, 400x). Images are representative of one uninfected and six infected mice.

**Extended Data Fig. 4:**
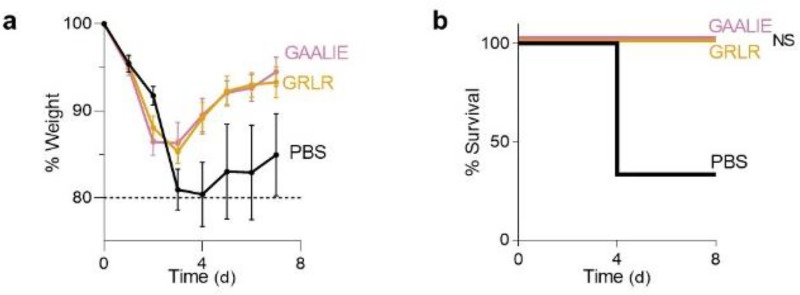
High-dose treatment of SARS-CoV-2-infected FcγR humanized mice with anti-SARS-CoV-2 mAbs Fc variants enhanced for activating FcγR binding is not associated with enhanced disease. Following the experimental strategy in Fig. 1b, SARS-CoV-2-infected (MA10, 10^4^ pfu, i.n.) FcγR humanized mice (n=3 for PBS and n=5 for mAb-treated groups in one experiment) were treated (i.v.) with 40 mg/kg REGN mAb cocktail expressed as Fc variants with diminished (GRLR) or enhanced activating FcγR binding (GAALIE). Comparison of **a**, Weight loss (mean ± s.e.m.) and **b**, survival was compared between GRLR and GAALIE-treated groups by two-way ANOVA (Bonferroni post hoc analysis adjusted for multiple comparisons) and log-rank (Mantel–Cox) test, respectively. NS, not significant.

**Extended Data Fig. 5:**
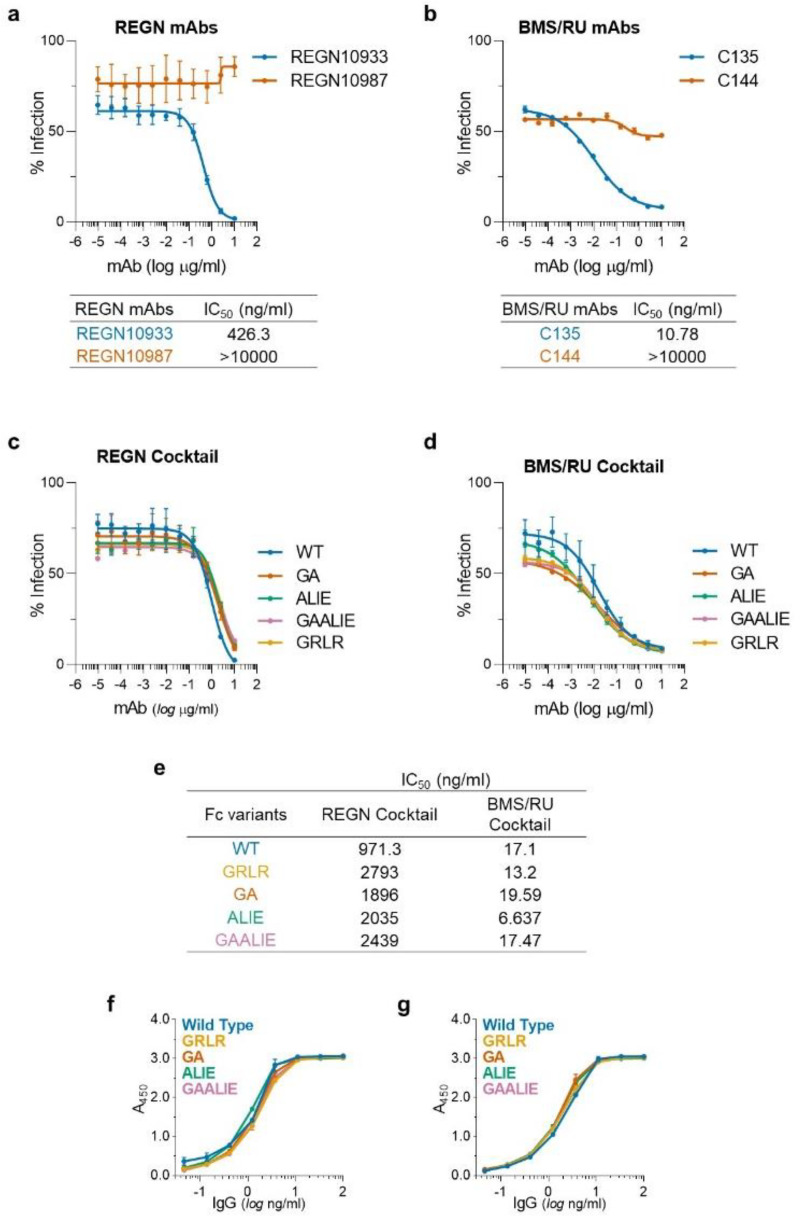
*In vitro* neutralization activity and antigenic specificity of Fc variants of anti-SARS-CoV-2 mAbs. To confirm that changes in the Fc domain have no effect on the neutralization activity and Fab-mediated functions of anti-SARS-CoV-2 mAb, Fc domain variants were characterized in (**a**-**e**) *in vitro* neutralization assays using SARS-CoV-2 (MA10) pseudotyped reporter viruses and (**f**, **g**) in ELISA assays using SARS-CoV-2 RBD. n= 1 experiment performed in duplicates. **a**, **b**, *In vitro* neutralization curves and IC_50_ values of REGN (**a**) and BMS/RU (**b**) mAbs against SARS-CoV-2 MA10. (**c**, **f**) REGN and (**d**, **g**) BMS/RU mAb cocktails were expressed as Fc variants and their *in vitro* neutralization activity (**c**, **d**, **e**, IC_50_ values) and antigenic specificity (**f**, **g**) was compared among Fc variants.

**Extended Data Fig. 6:**
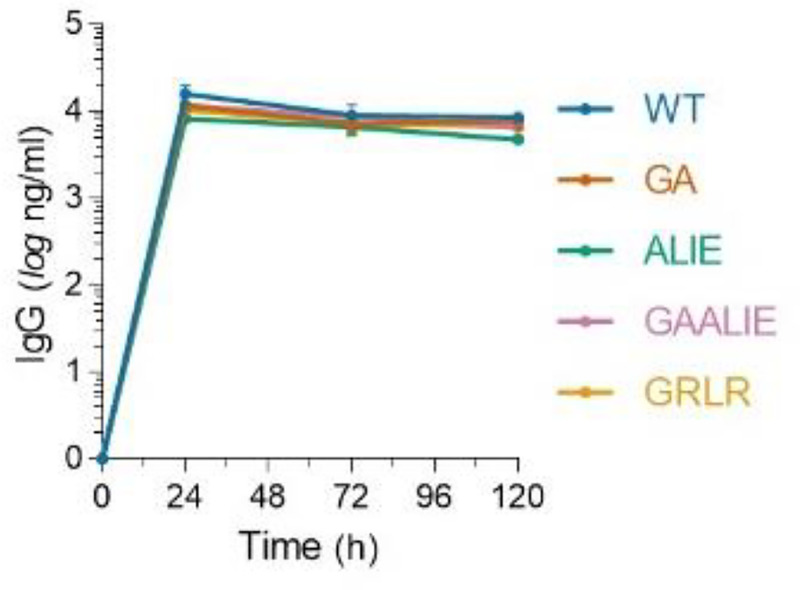
*In vivo* half-life of Fc variants of anti-SARS-CoV-2 mAbs. Fc variants of the REGN mAb cocktail were administered (i.v.; 50 μg) to FcγR humanized mice and antibody serum levels were determined by ELISA at various time points after antibody administration. n=3 mice per group in two independent experiments. Data are mean ± s.e.m.

## Figures and Tables

**Fig. 1:**
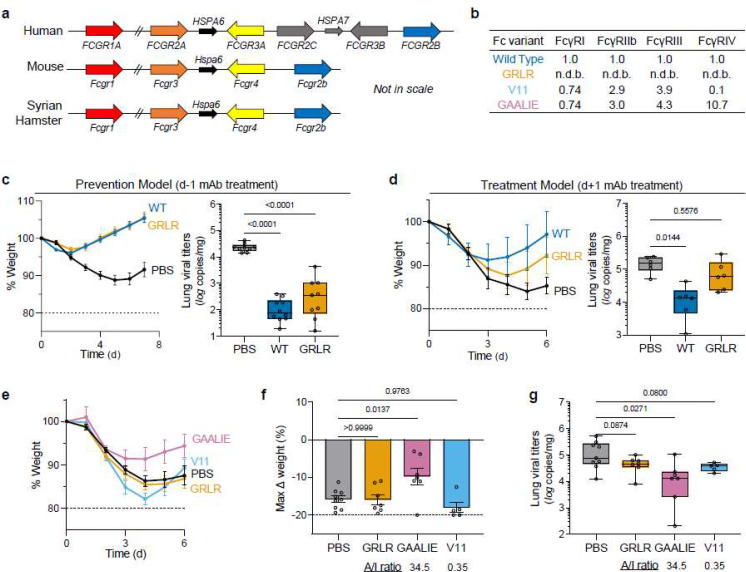
Contribution of Fc effector function to the protective activity of neutralizing anti-SARS-CoV-2 mAbs in hamster infection models. **a**, Overview of the FcγR locus organization in humans, mice, and Syrian hamsters. **b**, Fc variants of human IgG1 were evaluated for their affinity for hamster FcγRs. Numbers indicate the fold-change in affinity compared to wild-type human IgG1. n.d.b., no detectable binding. **c**, **d**, Wild-type and FcR null (GRLR) variants of REGN mAb cocktail (**c**) or S309 mAb (**d**) were administered i.v. (5 mg/kg) to Syrian hamsters one day before (prevention model, **c**) or after (therapy model, **d**) i.n. challenge with SARS-CoV-2 (NYC isolate, 10^5^ pfu) (n=9–10 hamsters per group from two independent experiments for **c** and n=6 hamsters per group from two independent experiments for **d**). Hamsters were monitored for weight loss (left; mean ± s.e.m.) and lung viral titers (right, analyzed on day 7 (**c**) or 6 (**d**) post-infection) were compared between treatment groups by one-way ANOVA (Bonferroni post hoc analysis adjusted for multiple comparisons). P values are indicated. **e**-**g**, SARS-CoV-2-infected hamsters (10^5^ pfu, NYC isolate) were treated on day 1 post-infection with Fc variants of the REGN mAb cocktail (5 mg/kg, i.v.) exhibit differential hamster FcγR binding affinity and A/I ratio (calculated based on FcγRIV/FcγRIIb affinity). Weight loss (**e**, plotted over time (mean ± s.e.m.) or **f**, as max change) and lung viral titers (**g**, assessed on day 6 post-infection) were compared by one-way ANOVA (Bonferroni post hoc analysis adjusted for multiple comparisons). P values are indicated. n=5–9 hamsters per group from two independent experiments.

**Fig. 2:**
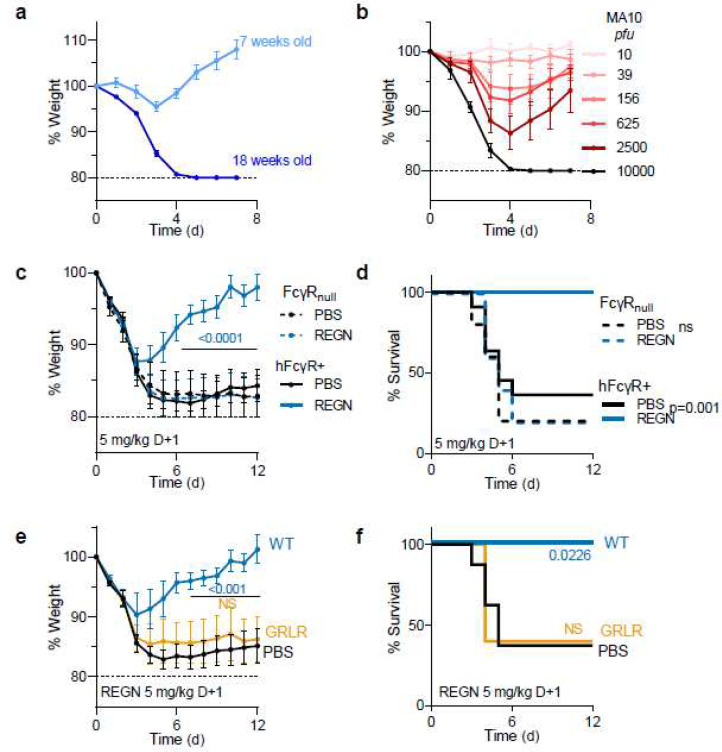
Fc-FcγR interactions are required for the therapeutic activity of neutralizing anti-SARS-CoV-2 mAbs in mouse infection models. **a**, **b**, FcγR humanized mice were infected with mouse-adapted SARS-CoV-2 (MA10 strain, 10^4^ pfu, i.n.) and weight loss (mean ± s.e.m.) was compared in (**a**) young (7 weeks old) and older (18 weeks old) mice, as well as in (**b**) mice (16–19 weeks old) challenged with the indicated inoculum dose. n=4–5 mice per group in two independent experiments. **c**, **d**, The therapeutic activity of REGN mAb cocktail (expressed as human IgG1 and administered at 5 mg/kg one day post-infection) was evaluated in FcγR humanized and FcγR deficient (FcγR_null_) mouse strains challenged with SARS-CoV-2 (MA10 strain, 10^4^ pfu i.n.). n=5 mice per group for FcγR_null_ and n=11–12 mice per group for FcγR humanized mice. **e**, **f**, SARS-CoV-2-infected FcγR humanized mice (MA10 strain, 10^4^ pfu i.n.) were treated with wild-type human IgG1 or GRLR variants of REGN mAb cocktail one day post-infection. n=5–8 mice per group in two independent experiments. Weight loss (**c**, **e**; mean ± s.e.m.) and survival curves (**d**, **f**) were compared to the corresponding PBS-treated group by two-way ANOVA (Bonferroni post hoc analysis adjusted for multiple comparisons) and log-rank (Mantel–Cox) test, respectively. P values are indicated. NS, not significant.

**Fig. 3:**
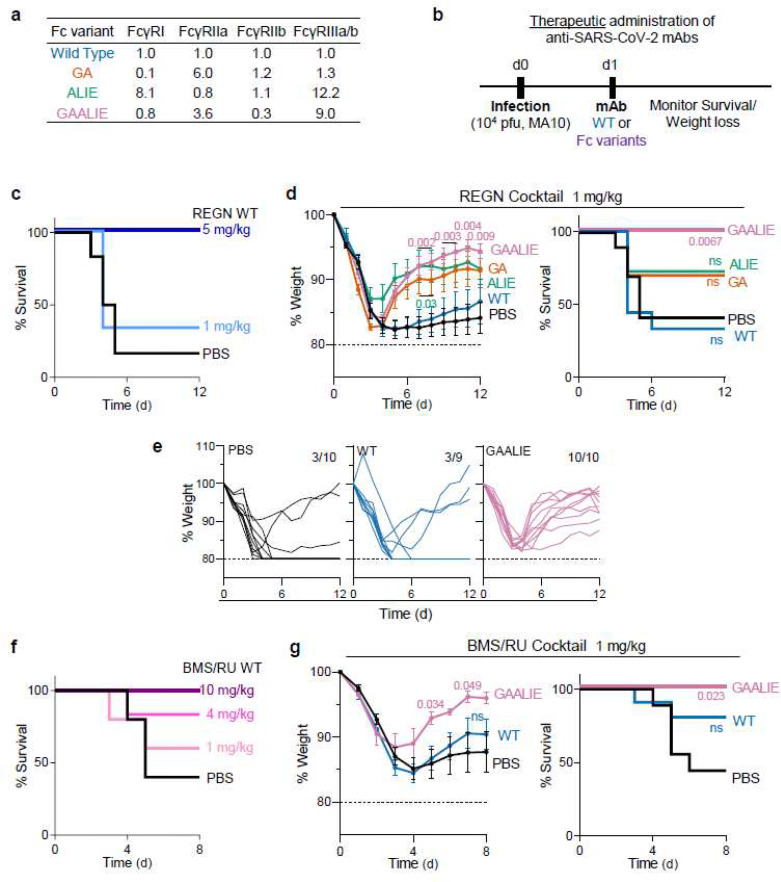
Selective engagement of activating FcγRs improves the therapeutic activity of anti-SARS-CoV-2 mAbs. **a**, Human IgG1 Fc variants with differential affinity for specific classes of human FcγRs were generated for anti-SARS-CoV-2 mAbs. Numbers indicate the fold-change in affinity compared to wild-type human IgG1. **b**-**g**, Following the experimental strategy in panel **b**, SARS-CoV-2-infected FcγR humanized mice were treated (i.v.) at the indicated dose with REGN (**c**-**e**) or BMS/RU (**f-g**) mAb cocktail expressed as wild-type human IgG1 or as Fc variants with differential affinity for human FcγRs. Weight loss (mean ± s.e.m.) (**d** and **g**, left panels; **e**, curves from individual mice) and survival curves (**c**, **f** and **d**, **g**, right panels) of antibody-treated mice were compared with the corresponding PBS-treated group by two-way ANOVA (Bonferroni post hoc analysis adjusted for multiple comparisons) and log-rank (Mantel–Cox) test, respectively. P values are indicated. NS, not significant. **c**, n=6 mice per group in two independent experiments; **d**, **e**, n=9–11 mice per group in four independent experiments; **f**, n=5–6 mice per group in one independent experiment; **g**, n=7–10 mice per group in two independent experiments.

**Fig. 4:**
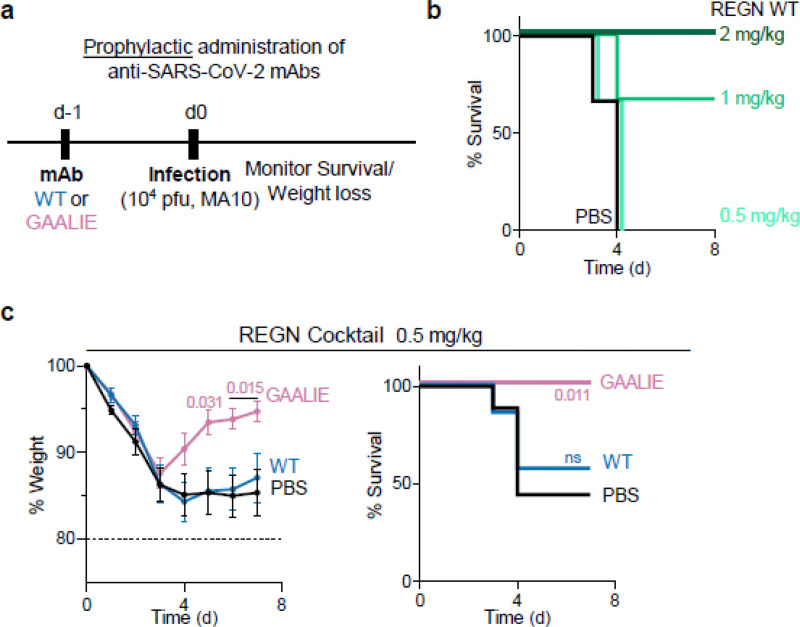
Prophylactic activity of anti-SARS-CoV-2 mAbs is enhanced by selective engagement of activating FcγRs. In a model of mAb-mediated prophylaxis of SARS-CoV-2 infection (**a**), the activity of wild-type and GAALIE variants of the REGN mAb cocktail was assessed. FcγR humanized mice were treated (i.v.) at the indicated dose with REGN mAb cocktail expressed as wild-type human IgG1 or as GAALIE variant one day prior to challenge with SARS-CoV-2 (MA10, 10^4^ pfu i.n.). Weight loss (mean ± s.e.m.) (**c**, left panel) and survival curves (**b** and **c**, right panel) of antibody-treated mice were compared with the PBS-treated group by two-way ANOVA (Bonferroni post hoc analysis adjusted for multiple comparisons) and log-rank (Mantel–Cox) test, respectively. P values are indicated. NS, not significant. **b**, n=3 mice per group in one experiment; **c**, n=7–9 mice per group in three independent experiments.
